# Computer Game Addiction and Loneliness in Children

**Published:** 2018-10

**Authors:** Hülya KÖK EREN, Özlem ÖRSAL

**Affiliations:** 1. Dept. of Psychiatric Nursing, Faculty of Health Sciences, Eskişehir Osmangazi University, Eskişehir, Turkey; 2. Dept. of Public Health Nursing, Faculty of Health Sciences, Eskişehir Osmangazi University, Eskişehir, Turkey

**Keywords:** Dependency, Loneliness, Child

## Abstract

**Background:**

We aimed to determine the level of computer game addiction and loneliness among 9–10-yr-old children.

**Methods:**

The study was conducted with 4^th^-grade students at a primary school, located at the city center, during 2017–2018 academic years. There was no sampling in the research, all 4^th^-grade students of the school were reached. “Personal Information Form”, “Computer Game Addiction Scale” and “UCLA Loneliness Scale” were used for collecting data. Mann Whitney U test, Kruskal Wallis test, and Correlation Analysis were used to evaluate the data of the research.

**Results:**

50.7% (n=104) of the students were female, most frequent number of sister/brother was one 39.0% (n=80), both their mother 31.7% (n=65) and their father 34.1% (n=69) were mostly high school graduated. The average scores that students got from the scales were; 48.66±.27.02 (min.: 21.00, max.: 105) for “Computer Game Addiction Scale” and 40.55±8.50 (min: 22.00, max.: 64) for “UCLA Loneliness Scale”. A weak, positive and significant relationship was found between students’ loneliness scale scores and computer game addiction scale scores (r=0.357; *P*<0.000).

**Conclusion:**

A significant relationship was found between students’ computer game addiction and loneliness. It is suggested to perform children’s loneliness and computer game addiction assessments, evaluate effectiveness and establish a rehabilitating treatment system among school-hospital-family for abnormal cases.

## Introduction

Nowadays, with the advancement of technology, the Internet has become indispensable to everybody. People use the internet for various purposes such as searching for information, gaming, listening to music, browsing the web, checking e-mail. In particular, the time that children and young adults constantly spend on the internet led to inappropriate use of the internet and caused problems such as internet and computer game addiction (1–3).

Third research appendix of Diagnostical and Statistical Manual of Mental Disorders-5 (DSM-5) addressed computer game addiction as internet gaming disorders. APA stated more requirements needed for supporting clinical diagnosis and defining it as a mental problem to add this disorder to the manual (4).

As in other addictions, game addiction causes mental and social symptoms on the individual (5, 6). The social development of the children, who widely use internet and spending this time with computer games, has slowed significantly, the self-confidence of these children is low (7), whereas their social anxiety level and aggression behavior were found to be high (8). There was a negative relationship between self-confidence and computer games (9). Computer games caused aggression in children and adolescents (10).

Loneliness is defined by Peplau and Perlman as an unpleasant mental state when the difference between the individual's existing social relationship and the social relationship that he/she wants to be in is recognized. The loneliness is an indication that there are major shortcomings in the relations of the individual and his/her insufficiency of skills in interpersonal relations. Established successful relationships with their peers is an important indicator of children’ social competence (11). The 9–10-yr-old children begin socializing during this period, give importance to learning and school success. Excessive Internet usage will prevent child’s socialization, will cause difficulty in face to face communication alone (12, 13). In the light of this information, the identification of the relationship between children’s game addiction and loneliness gains importance.

This study was conducted to determine the level of computer game addiction and loneliness in children.

## Methods

This descriptive nature study was conducted to determine the level of computer game addiction and loneliness of the children. The study was conducted with 4^th^-grade students at a primary school, located at the city center, during 2017–2018 academic years. There was no sampling in the research, all 4^th^-grade students of the school were reached (n=205).

### Data Collection Tools

“Personal Information Form”, “Computer Game Addiction Scale” and “UCLA Loneliness Scale” were used for collecting data.

*Personal Information Form:* The personal information form developed for this research includes a total of nine questions such as gender, number of brother/sister, education level of the parents, number of hours spent playing games each day.

*Computer Game Addiction Scale:* This scale was developed by Horzum, Ayas and Balta (2008). It has 4-factors structure, including a total of 21 items. The first factor of the scale, called as “*being unable to stop playing computer game*”, consists of ten items. The second factor of the scale, called as “*associating computer game with reel life”* consists of four items. Internal consistency coefficient of this factor is 0.60. The third factor of the scale consists of three items, called as “*neglecting the duties due to playing computer games*” and internal consistency coefficient of this factor is 0.50. The last factor of the scale, called as “*preferring playing computer game to other activities*”, consists of four items. Internal consistency coefficient of this factor is 0.50. Considering 21-items scale as a whole, it explains 45% of total variance and its internal consistency coefficient is 0.85. Minimum score that can be taken from the scale is 21, whereas the maximum is 105. All the items covered by the scale are positive expressions (14). Cronbach Alfa internal consistency coefficient of the scale, calculated for the sample of our study, is 0.82.

*UCLA Loneliness Scale (UCLA-LS):* This is a 20-items scale developed for determining loneliness level (15). Higher scores mean more intense loneliness. Validity and reliability study of the Turkish scale was performed (16). Cronbach Alfa internal consistency coefficient was found to be 0.96, whereas test-retest reliability was 0.94. Cronbach Alfa internal consistency coefficient of the scale, calculated for the sample of our study is 0.79.

### Application of Data Collection Tools

Before the application of the data collection forms, the approval of Provincial Directorate of Education and Eskişehir Osmangazi University Ethics Committee (Dated 26.09.2017, numbered 80558721/ 260) were granted.

The data were collected by the researcher, in Nov–Dec 2017, during the lectures determined before. The parents were informed about the subject and objective of the study and written approval of the parents, who accepted the participation of their children, were taken. Then, students were informed about the subject and objective of the study.

### Evaluation of the Data

Frequency tables and descriptive statistics were used for the interpretation of the data. In accordance with non-parametric methods, Mann-Whitney U Test was used to compare the scores of 2 independent groups, whereas Kruskal-Wallis H Test was employed to compare the scores of 3 or more independent groups. The data obtained from the scales did not fit normal distribution, therefore the analysis concerning the direction, degree and significance of the relationship between the scales were performed using Spearman Correlation method.

## Results

50.7% (n=104) of the students were female, most frequent number of sister/brother was one 39.0% (n=80), most of the parents were mostly high school graduated (mother 31.7% (n=65) and their father 34.1% (n=69)) (Table 1). 34.6% (n= 71) of the students played game 30 min/day, whereas 25.4% (n=52) played 120 min/day. 44.4% (n= 91) of the students were rarely rejecting to sleep for playing games, 58.5% (n= 120) got aggressive when their playtime was reduced or restrained. 61.0% (n= 1205) of the students always encountered difficulties in case of exaggerating to play game (Table 2). The average scores that students got from the scales were 48.66±.27.02 (min: 21.00, max.: 105) for “Computer Game Addiction Scale” and 40.55±8.50 (min: 22.00, max.: 64) for “UCLA Loneliness Scale”.

**Table 1:** Comparison of Computer Game Addiction and Loneliness Scale by Descriptive Characteristics of Student

***Descriptive Characteristics***	***n***	***%***	***Factor 1 x̄ ± sd***	***Factor 2 x̄ ± sd***	***Factor 3 x̄ ± sd***	***Factor 4 x̄ ± sd***	***Game Addiction x̄ ± sd***	***Loneliness x̄ ± sd***
**Gender**								
*Male*	101	49.3	28.92±13.97	11.34±5.83	6.66±4.32	10.36±5.67	57.28±27.69	41.87±8.38
*Female*	104	50.7	19.23±12.08	8.18±5.28	4.77±3.50	8.13±5.03	40.31±23.65	39.27±8.46
**Test**			*Z=−4.826*	*Z=−3.996*	*Z=−4.048*	*Z=−3.188*	*Z=−4.312*	*Z=−2.237*
***P***			*P=0.000*	*P=0.000*	*P=0.000*	*P=0.001*	*P=0.000*	*P=0.025*
**Number of brother/sister**								
*One*	80	39.0	24.08±14.49	9.89±5.90	5.63±4.03	9.40±5.50	48.99±27.37	40.30±8.73
*Two*	74	36.1	22.18±12.91	9.00±5.46	5.26±3.70	8.39±5.02	44.84±25.50	39.13±7.84^a^
*Three and over*	51	24.9	26.53±14.18	10.57±5.95	6.47±4.44	10.16±5.92	53.73±28.27	43.02±8.70^a^
**Test**			*X*^*2*^=1.994	*X*^*2*^=1.765	*X*^*2*^=2.374	*X*^*2*^=2.454	*X*^*2*^=2.276	*X*^*2*^=7.050
***P***			*P*=0.369	*P*=0.414	*P*=0.305	*P*=0.293	*P*=0.320	*P*=0.029
**Mother's education level**								
*Primary school*	57	27.8	22.14±13.66	8.91±5.54	5.14±3.75	8.44±5.64	44.63±26.75	42.33±8.79
*Secondary school High school University*	59	28.8	25.02±13.78	9.95±5.97	5.88±4.22	9.84±5.56	50.69±27.64	40.32±9.26
	65	31.7	25.60±14.47	10.60±5.95	6.37±4.34	9.63±5.42	52.20±27.68	39.18±7.79
	24	11.7	21.63±13.10	8.83±5.18	4.79±3.06	8.46±4.84	43.71±23.78	40.43±7.42
**Test**			*X*^*2*^=3.557	*X*^*2*^=3.286	*X*^*2*^=3.886	*X*^*2*^=4.112	*X*^*2*^=4.206	*X*^*2*^=4.301
***P***			*P*=0.313	*P*=0.350	*P*=0.274	*P*=0.250	*P=*0.240	*P*=0.231
**Father’s education level**								
*Primary school*	22	10.7	24.91±15.47	9.86±6.62	6.27±4.76	9.73±6.10	50.77±32.14	41.23±9.15
*Secondary school High school University*	44	21.5	27.14±14.63	10.68±5.93	6.81±4.44	10.39±6.42	55.02±29.34	44.13±8.59^a.b^
	70	34.1	22.19±12.82	9.00±5.28	5.20±3.64	8.47±4.91	44.86±24.57	40.07±7.61^a^
	69	33.7	23.57±13.88	9.84±5.87	5.32±3.80	9.09±5.09	47.81±25.95	38.55±8.53^b^
**Test**			*X*^*2*^=3.070	*X*^*2*^=1.886	*X*^*2*^=5.085	*X*^*2*^=1.910	*X*^*2*^=2.630	***X*^2^=12.827**
***P***			*P*=0.381	*P*=0.596	*P*=0.166	*P*=0.591	*P*=0.452	***P*=0.005**

**Table 2:** Comparison of Computer Game Addiction and Loneliness Scale by Students Game Play Features

***Game Play Features***	***n***	***%***	***Factor 1 x̄ ± sd***	***Factor 2 x̄ ± sd***	***Factor 3 x̄ ± sd***	***Factor 4 x̄ ± sd***	***Game Addiction x̄ ± sd***	***Loneliness x̄ ± sd***
**Daily Gaming**								
**Time**								
*Hiç*	25	12.2	22.12±14.77	9.00±5.79	5.40±4.16	8.60±5.45	45.12±28.28	38.92±9.28
*30 Min.*	71	34.6	21.63±12.52	9.07±5.38	5.31±3.75	9.01±5.18	45.03±24.70	40.04±8.90
*60 Min.*	46	22.4	23.11±13.92	9.43±5.72	5.06±3.65	8.63±5.56	46.24±26.98	40.76±8.78
*90 Min.*	11	5.4	27.90±15.52	11.00±7.20	6.45±4.80	10.91±5.80	56.27±30.97	37.27±8.32
*120 Min.*	52	25.4	28.11±14.32	11.00±5.94	6.79±4.39	9.98±5.73	55.88±27.96	42.56±7.08
**Test**			X^2^=9.058	X^2^=5.435	X^2^=8.521	X^2^=4.285	X^2^=8.151	X^2^=5.720
***P***			*P*=0.060	*P*=0.246	*P*=0.074	*P*=0.369	*P*=0.086	*P*=0.221
**Rejecting to sleep for playing games**																
*Never*	56	27.3	15.48±10.00^a.b.c^	6.45±4.45^a.b.c^	4.07±2.95^a.b.c^	6.84±4.72^a.b.c^	32.84±19.91^a.b.c^	37.27±8.08^a.b^
*Rarely*	91	44.4	23.24±12.55^a.d.e^	9.62±5.41^a.d^	4.86±3.09^a.d.e^	8.85±5.02^a.d^	46.57±23.84^a.d.e^	40.07±8.42^c^
*Sometimes*	34	16.6	30.74±14.12^b.d^	12.26±6.10^b^	7.52±4.89^b.d^	11.00±5.66^b^	61.53±28.33^b.d^	43.85±8.08^a^
*Always*	24	11.7	37.25±11.87^c.e^	14.29±4.68^c.d^	10.12±4.12^c.e^	13.67±5.18^c.d^	75.33±23.25^c.e^	45.42±6.79^b.c^
**Test**			*X*^*2*^*=51.381*	*X*^*2*^*=44.431*	*X*^*2*^*=49.040*	*X*^*2*^*=33.548*	*X*^*2*^*=50.076*	X^2^=23.631
***P***			*P=0.000*	*P=0.000*	*P=0.000*	*P=0.000*	*P=0.000*	*P*=0.000
**Emotions felt when students’ computer games play is reduced or restrained**																
*Anxious*	31	15.1	33.32±13.19^a^	12.71±5.39^a^	7.94±4.59^a^	11.61±5.59^a^	65.85±25.95^a^	43.51±7.55^a^
*Unhappy*	33	16.1	27.73±12.22^b^	12.00±5.86^b^	6.70±4.21^a^	10.42±5.24^b^	56.85±24.21^b^	42.61±9.54
*Miss playing the game*	21	10.2	31.81±12.54^c^	12.81±5.26^c^	6.33±4.12^c^	11.14±5.50^c^	62.09±25.52^c^	39.67±7.63
*Angry*	120	58.5	19.21±12.65^a.b.c^	7.81±5.16^a.b.c^	4.74±3.51^a.b.c^	7.94±5.15^a.b.d^	39.70±24.68^a.b.c^	39.87±8.39^a^
**Test**			*X*^*2*^*=39.835*	*X*^*2*^*=36.618*	*X*^*2*^*=23.035*	*X*^*2*^*=20.230*	*X*^*2*^*=38.873*	X^2^=8.213
***P***			*P=0.000*	*P=0.000*	*P=0.000*	*P=0.000*	*P=0.000*	*P*=0.042
**Difficulties that students encounter with their surroundings**								
*Never*	125	61.0	19.54±12.73^a.b^	8.11±5.22^a.b^	4.43±3.13^a.b^	7.60±4.74^a.b^	39.69±23.28^a.b^	37.99±8.21^a^
*Sometimes*	63	30.7	29.97±12.04^b^	11.75±5.41^b^	7.29±4.28^b^	11.28±5.37^b^	60.29±24.89^b^	44.84±7.32^b^
*Always*	17	8.3	34.71±15.04^a^	14.23±6.47^a^	9.18±4.95^a^	13.53±6.10^a^	71.65±31.43^a^	43.53±7.66^a.b^
**Test**			*X*^*2*^*=35.346*	*X*^*2*^*=27.420*	*X*^*2*^*=37.333*	*X*^*2*^*=30.239*	*X*^*2*^*=36.034*	X^2^=29.732
***P***			*P=0.000*	*P=0.000*	*P=0.000*	*P=0.000*	*P=0.000*	*P*=0.000

### Comparison of Computer Game Addiction and Loneliness Scale by Descriptive Characteristics of Students

There were significant differences among the overall and all sub-factor scores of Computer Game Addiction Scale according to gender, where the score of the males was significantly higher than females (*P*<0.01) (Table 1).

There was no statistically significant difference in terms of factor scores taken from the point of view of number of brother/sister, education of mother and education of father. There were statistically significant differences between the score of loneliness scale according to gender (*P*=0.025). The average score that males obtained from loneliness scale were greater than females. There were statistically significant differences between the score of loneliness scale according to the number of brother/sister (*P*=0.029).

The average score of the students with two brother/sister was lower than those who had three or more brother/sister. There were statistically significant differences between the score of loneliness scale according to the education level of the father (*P*=0.005). The average score of the students whose father’s education level was secondary school was higher than those whose father’s education level was high school and university (Table 1).

### Comparison of Computer Game Addiction and Loneliness Scale by students Gameplay Features

There were statistically significant differences between the overall and all sub-factor scores of Computer Game Addiction Scale according to rejecting to sleep for playing games. The overall and sub-dimensions average scores of the students who always reject to sleep was higher than those who rarely and never rejected to sleep (*P*<0.01). There were also statistically significant differences in terms of overall scale and all sub-factors according to the emotions felt when students’ computer games playtime was reduced or restrained. Accordingly, the overall and sub-dimensions scores of the students who get angry was significantly lower than those who get upset, anxious and who misses the game (*P*<0.01). As for students’ experience with their surroundings, there were statistically significant differences in the overall and all factors scores of computer game addiction scale. The scores of the students who always encounter problem with their surroundings when they play computer games were higher than the students who never experience problems with their surroundings (*P*<0.01) (Table 2).

There were statistically significant differences among the scores of loneliness scale according to rejecting to sleep for playing games (*P*<0.01). The overall loneliness scale scores of the students who always reject to sleep were higher than those who rarely and never reject to sleep. Their scores of the scale statistically differentiate according to the emotions felt when students’ computer games play was reduced or restrained (*P*=0.042). The loneliness score of the students who get anxious was higher than those who get angry. Regarding the difficulties that students encounter with their surroundings, there were statistically significant differences in loneliness scale scores (*P*<0.01). The scores of the students who always encounter problem were higher than the students who never or sometimes experience problems (Table 2).

### Correlation between Points Taken From Students' Loneliness Scale and Points Taken From Computer Game Addiction Scale

A weak, positive and significant relationship was found between students’ loneliness scale scores and computer game addiction scale scores (r=0.357; *P*<0.01).

## Discussion

According to the outcomes of our study, performed to determine the level of computer game addiction and loneliness among children, computer game addiction scores for the males are higher than females. Addiction level of the male students was reported to be higher than female students in many studies (7, 17, 18). The reason behind it may be the fact that females and males use computer for different purposes. Females spend more time messaging and surfing across websites, whereas males use computer for game playing purpose (19). 67.1% of the males, 18,9% of the females play computer games more than 5 h per day (2).

In our study, overall and all sub-dimensions scores of the students who always reject to sleep are higher than those who never or rarely reject to sleep. Adolescents who play game on internet more than four hours have sleeping difficulties, whereas another study revealed that 26,7% of the youngsters who are addictive to internet experience sleeping problems (20, 21). Another research conducted with the children revealed that sleep deprivation is a preceding demonstrative of Internet addiction (22).

The average overall and sub-dimensions scores of the students, who get angry when their computer game play is reduced or restrained, is lower than those who get upset, anxious and who miss the game. Psychiatrist Ivan Goldberg has accepted Internet addiction as a disorder in 1999 and developed full criteria (23). According to these criteria, one of the symptoms of internet addiction is feeling anxiousness, depression in case of reducing or completely restraining internet connection.

The overall and all sub-dimensions scores of the students, who always experience problem with their surroundings when they play computer games, are higher than the students who never encounter problems with their surroundings. According to “Insufficient Social Skills” theory that Caplan has developed for addiction, individuals whose social relations with others are insufficient are more likely to be addictive (24).

In our study, the score of loneliness increases as the score of computer game addiction increases. The studies conducted with adolescents revealed a significant relationship between game addiction and loneliness (25–27). The result of pathological game addiction is loneliness (28). The 10-yr monitoring study conducted with young adults revealed that game addictive cannot establish close friendship (29).

## Conclusion

The game addiction is high among male students, those who get angry when computer game play is reduced or restrained, who always experience problems with their surroundings when they play computer games. Our study revealed a significant relationship between students’ game addiction and loneliness, in other words, as game addiction increases, loneliness increases. It is suggested to perform children’s loneliness and computer game addiction assessments, evaluate them effectively and establish a rehabilitating treatment system among school-hospital-family for abnormal cases.

## Ethical considerations

Ethical issues (Including plagiarism, informed consent, misconduct, data fabrication and/or falsification, double publication and/or submission, redundancy, etc.) have been completely observed by the authors.
